# Magnetic nanoparticle supported hyperbranched polyglycerol catalysts for synthesis of 4*H*-benzo[*b*]pyran

**DOI:** 10.1007/s00706-013-1026-3

**Published:** 2013-07-12

**Authors:** Mohammad Ali Nasseri, Seyed Mohsen Sadeghzadeh

**Affiliations:** Department of Chemistry, College of Sciences, Birjand University, PO Box 97175-615, Birjand, Iran

**Keywords:** Magnetic nanoparticle, 4*H*-Benzo[*b*]pyran, Solvent-free, One-pot synthesis, Green chemistry

## Abstract

**Abstract:**

A magnetic nanoparticle supported hyperbranched polyglycerol catalyst was prepared readily from inexpensive starting materials in aqueous medium that catalyzed the synthesis of 4*H*-benzo[*b*]pyran under solvent-free conditions at room temperature. X-ray diffraction, transmission electron microscopy, thermal gravimetric analysis, vibrating sample magnetometry, and selected-area electron diffraction were employed to characterize the properties of the synthesized catalyst. Its high catalytic activity and ease of recovery from the reaction mixture using an external magnet, and the possibility of reusing several times without significant loss of performance are additional eco-friendly attributes of this catalytic system.

**Graphical Abstract:**

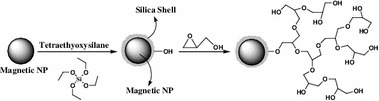
.

## Introduction

In recent years, core–shell multi-components have attracted intense attention because of their potential applications in catalysis [1]. Unlike single-components that can supply only one function, core–shell multi-components can integrate multiple functions into one system for specific applications [2–6]. Moreover, the interactions between different components can greatly improve the performance of the multi-component system and even generate new synergetic properties. Among core–shell structured composites, those with a magnetic core and functional shell structures have received special attention because of their potential applications in catalysis, drug storage/release, selective separation, chromatography, and chemical or biologic sensors [7–12]. The magnetic core has good magnetic responsiveness, and can be easily magnetized. Therefore, composites with magnetic cores can be conveniently collected, separated, or fixed using an external magnet.

4*H*-Benzopyran derivatives are a major class of heterocycles, and 4*H*-pyran derivatives have attracted strong interest due to their useful biological and pharmacological properties such as anticoagulant, spasmolytic, diutretic, anticancer [13], and antianaphylactin characteristics [14]. 4*H*-Pyrans also occur in various natural products [15] and some benzopyran derivatives have been reported to have photochemical activities [16]. Development of 4*H*-pyran synthesis has been of considerable interest in organic synthesis, because of their wide-ranging biological and pharmaceutical activities. Consequently, numerous methods for the synthesis of 4*H*-pyrans have been reported. A variety of reagents, such as Yb(PFO)_3_ [17], tetramethylammonium hydroxide [18], Na_2_SeO_4_ [19], LiBr [20], NaBr [21], MgO [22], SB-DABCO [24], and the use of microwave irradiation [23], were found to catalyze these reactions. However, some of the reported methods have the following drawbacks: use of expensive reagents, long reaction times, low product yields, and use of an additional microwave oven. Herein we report the fabrication of hyperbranched polyglycerol (HPG) incorporated into mesoporous magnetite nanoparticles (MNP) that catalyze the synthesis 4*H*-benzo[*b*]pyrans under solvent-free conditions at room temperature (Scheme 1).

**Figure:**
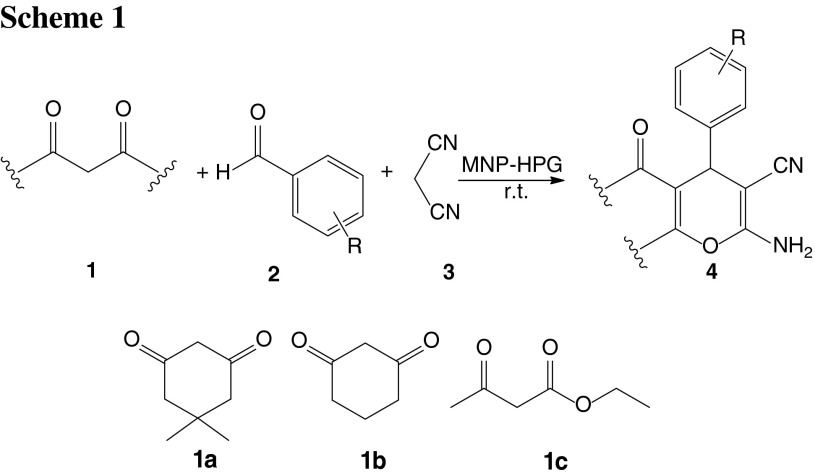

## Results and discussion

**Fig. 1:**
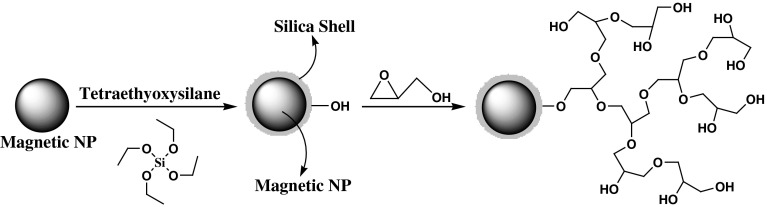
Schematic illustration of the synthesis for magnetic nanoparticle supported hyperbranched polyglycerol (MNP-HPG)

We report the synthesis of a magnetic particle-based solid polymer with a high density of HPG groups and discuss its performance as a novel strong and stable solid polymer. We were intrigued by the possibility of applying anhydrous dioxane and nanotechnology to the design of a novel, active, recyclable, and magnetically recoverable HPG derivative for the first time (Fig. 1).

Normally, N_2_H_4_·H_2_O can serve as either an oxidant or a reducer in alkaline solution. Ni^2+^ can be reduced easily to Ni in alkaline solution by N_2_H_4_·H_2_O. However, it is difficult to reduce Fe^2+^ to Fe directly by N_2_H_4_·H_2_O because the electromotive force of the oxidation reaction of Fe^2+^ to Fe^3+^ (0.66 V) is much larger than that of the reduction reaction from Fe^2+^ to Fe (0.283 V). Thus, Fe^2+^ is more likely to be oxidized to Fe^3+^ when treated by N_2_H_4_·H_2_O in alkali solution. In this experiment, however, when Fe^2+^ and Ni^2+^ coexist in the solution, Fe^2+^ ions can be reduced easily to Fe under the assistance of Ni^2+^ to form FeNi_3_ alloy. The reduction reaction can be expressed as follows:

$$ {3\text{Ni}^{2+}+\text{Fe}^{2+}+2\text{N}_2\text{H}_4+8\text{OH}^{-} \rightarrow \text{FeNi}_3+2\text{N}_2+8\text{H}_2\text{O}}. $$

 

### X-ray power diffraction

**Fig. 2:**
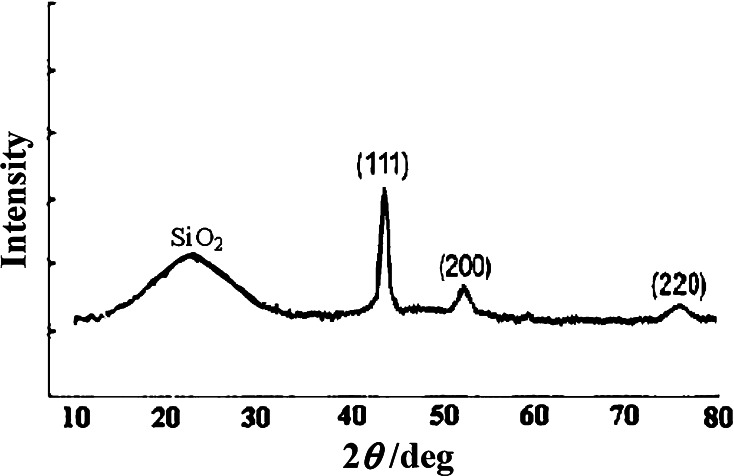
X-ray power diffraction (XRD) analysis of MNP-HPG

The structural properties of synthesized FeNi_3_/SiO_2_/HPG nanoparticle were analyzed by X-ray power diffraction (XRD). As shown in Fig. 2, the XRD pattern of the synthesized FeNi_3_/SiO_2_/HPG nanoparticle displays several relatively strong reflection peaks in the 2*θ* region of 40°–80°, which is quite similar to those of FeNi_3_ nanoparticles reported by other groups. Three characteristic peaks for FeNi_3_ (2*θ* = 44.3°, 51.5°, 75.9°) from (111), (200), and (220) planes were obtained. In addition, no iron and nickel oxides or other impurity phases were detected in the XRD patterns. The sharp and strong diffraction peaks confirm the good crystallization of the products. The broad band at 2*θ* = 15.0°–30.0° can be assigned to the amorphous SiO_2_ shell (JCPDS No. 29-0085).

### High-resolution transmission electron microscopy

**Fig. 3:**
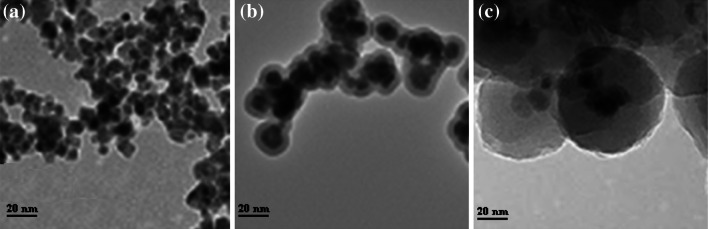
High-resolution transmission electron microscopy (HRTEM) images of **a** FeNi_3_, **b** FeNi_3_/SiO_2_, and **c** FeNi_3_/SiO_2_/HPG

High-resolution transmission electron microscopy (HRTEM) images of FeNi_3_, FeNi_3_/SiO_2_, and FeNi_3_/SiO_2_/HPG MNPs are shown in Fig. 3. The average size of FeNi_3_ MNPs is about 15 nm, and the aggregation of the nanoparticles can be discerned clearly (Fig. 3a). After being coated with a silica layer, the typical core–shell structure of the FeNi_3_/SiO_2_ MNPs can be observed. The dispersity of FeNi_3_/SiO_2_ MNPs is also improved, and the average size increases to about 20 nm (Fig. 3b). The average size of FeNi_3_/SiO_2_/HPG MNPs is about 60 nm (Fig. 3c), but aggregation of FeNi_3_/SiO_2_/HPG is more evident than that of FeNi_3_/SiO_2_ MNPs.

### Selected-area electron diffraction

**Fig. 4:**
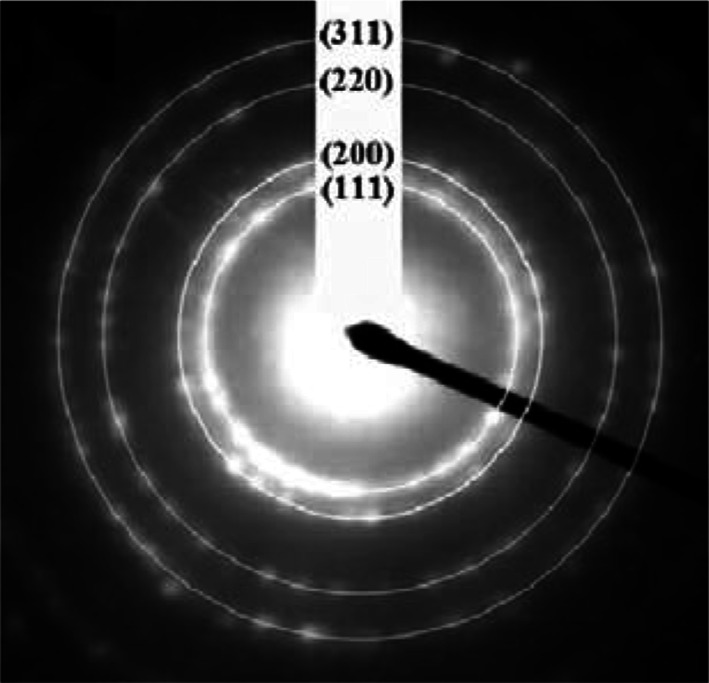
Selected-area electron diffraction (SAED) pattern of FeNi_3_/SiO_2_/HPG MNP

The selected-area electron diffraction (SAED) pattern taken from the prepared FeNi_3_/SiO_2_/HPG MNPs consists of typical polycrystalline rings, suggesting a nanocrystalline structure (Fig. 4). The diffraction peaks from (111), (200), (220), and (311) planes of (FCC)-FeNi_3_ are in total agreement with those of XRD.

### Thermogravimetric analysis

**Fig. 5:**
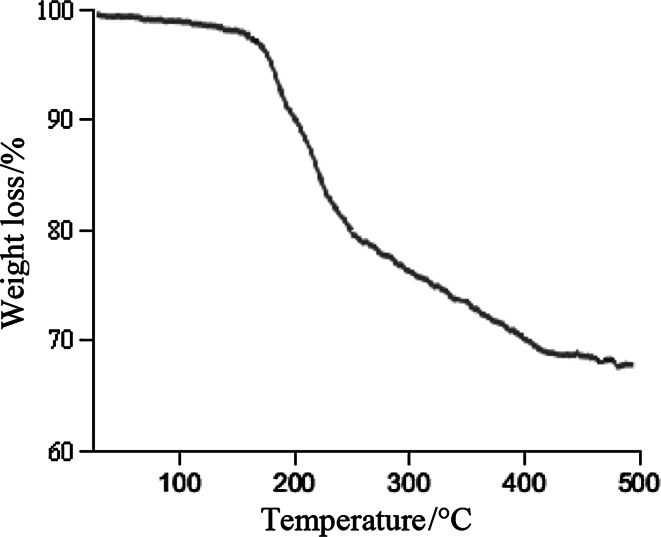
Thermogravimetric analysis (TGA) of FeNi_3_/SiO_2_/HPG MNP

The thermal behavior of FeNi_3_/SiO_2_/HPG MNPs (Fig. 5) was evaluated to be 1.5 % according to thermogravimetric analysis (TGA). The analysis showed two decreasing peaks. The first peak appears at temperature around 130–150 °C due to desorption of water molecules from the catalyst surface. This is followed by a second peak at 425–450 °C, corresponding to the loss of the organic spacer group.

### Magnetic properties of FeNi_3_/SiO_2_/HPG MNP

**Fig. 6:**
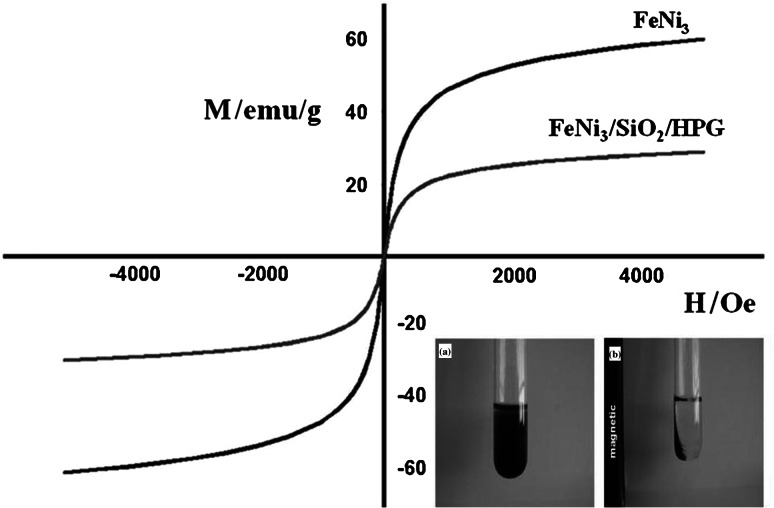
Room temperature magnetization curves of the FeNi_3_ MNP and FeNi_3_/SiO_2_/HPG MNP

The magnetization curves of FeNi_3_ and FeNi_3_/SiO_2_/HPG MNPs were further recorded at room temperature (Fig. 6). The magnetizations were expressed in units of emu per gram of powder. The two measured samples display a superparamagnetic behavior, as evidenced by a zero coercivity and remanence on the magnetization loop. The saturation magnetization value of the FeNi_3_/SiO_2_/HPG MNP is 25 emu/g, which is lower than that of uncoated magnetic particles (about 60 emu/g).

### Catalytic activity of FeNi_3_/SiO_2_/HPG MNPs

The effect of solvent on this reaction was examined and the results obtained are summarized in Table 1. In *n*-hexane, CHCl_3_, and dioxane (Table 1, entries 12–14), only a trace of product was observed. On the contrary, moderate yields could be achieved in other solvents (Table 1, entries 1–10). More strikingly, we found that the reaction proceeded smoothly in solvent-free conditions and gave the desired product in 97 % yield (Table 1, entry 11).

**Table 1:** Solvent screening for the reaction between benzaldehyde, malononitrile, and dimedone

Entry	Solvent	Yield/%^a^
1	H_2_O	82
2	EtOH	76
3	CH_3_CN	57
4	THF	36
5	CH_2_Cl_2_	39
6	Toluene	17
7	EtOAC	72
8	MeOH	78
9	DMF	63
10	DMSO	67
11	Solvent free	97
12	*n*-Hexane	Trace
13	CHCl_3_	Trace
14	Dioxane	Trace

Reaction conditions: malononitrile (1 mmol), dimedone (1 mmol), benzaldehyde (1 mmol), and 0.001 g FeNi_3_/SiO_2_/HPG MNP at room temperature for 45 min

^a^Isolated yields

**Fig. 7:**
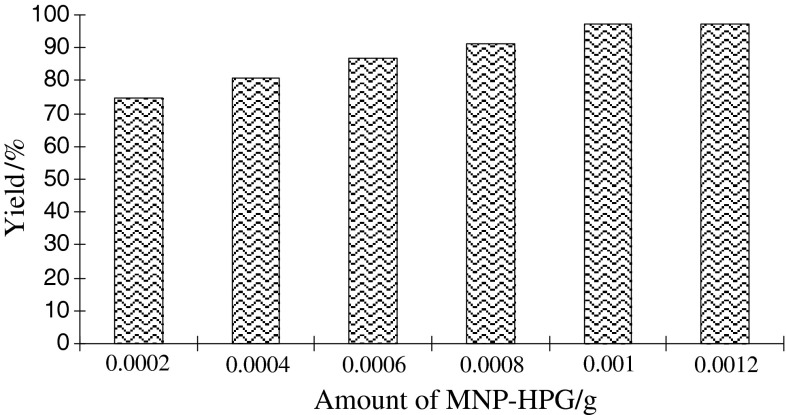
Effect of increasing amount of FeNi_3_/SiO_2_/HPG MNP on the preparation of 4*H*-benzo[*b*]pyran

At this stage, the amount of catalyst necessary to promote the reaction efficiently was examined. It was observed that variation of the amount of FeNi_3_/SiO_2_/HPG MNP had an effective influence. The best amount of FeNi_3_/SiO_2_/HPG MNP was 0.001 g, which afforded the desired product in 97 % yield (Fig. 7).

**Fig. 8:**
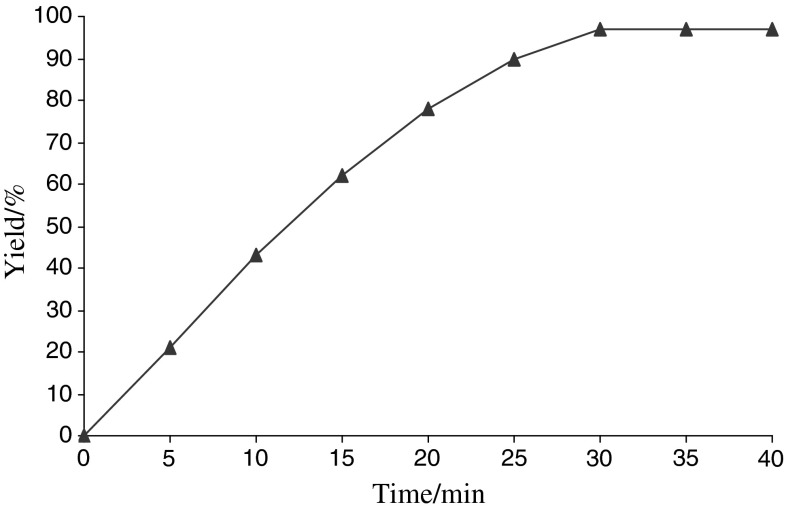
Reaction progress monitored by gas chromatography (GC). Reaction conditions: dimedone (1 mmol), benzaldehyde (1 mmol), malononitrile (1 mmol), and 0.001 g FeNi_3_/SiO_2_/HPG MNP at room temperature

Progress of the reaction in the presence of 0.001 g FeNi_3_/SiO_2_/HPG MNP was monitored by gas chromatography (GC) under optimal conditions (Fig. 8). Using this catalyst system, excellent yields of 4*H*-benzo[*b*]pyran can be achieved in 30 min. No apparent by-products were observed by GC in any of the experiments and the cyclic carbonate was obtained cleanly in 97 % yield.

**Fig. 9:**
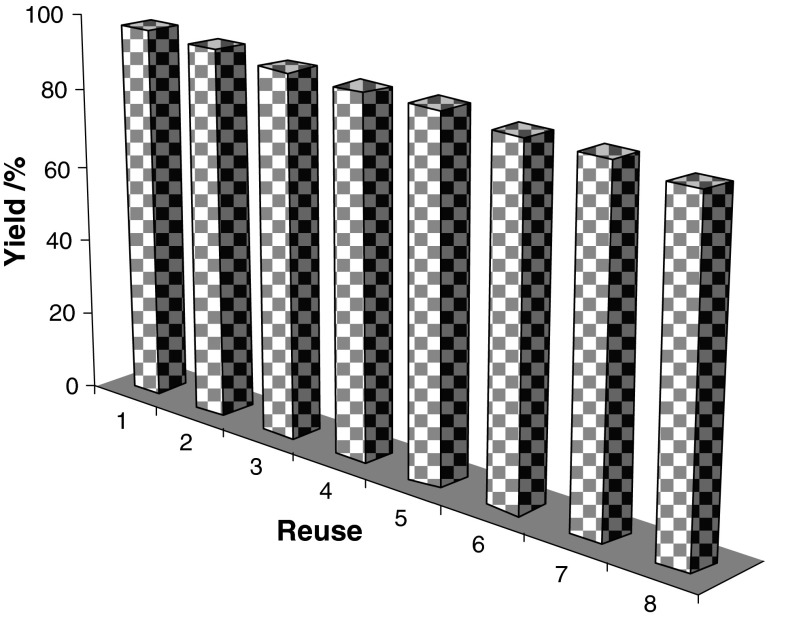
Reuse performance of the catalyst

It is important to note that the magnetic property of FeNi_3_/SiO_2_/HPG MNP facilitates its efficient recovery from the reaction mixture during work-up procedure. The activity of the recycled catalyst was also examined under the optimized conditions. After completion of the reaction, the catalyst was separated using an external magnet, washed with methanol and dried at the pump. The recovered catalyst was reused for eight consecutive cycles without any significant loss in catalytic activity (Fig. 9).

As can be seen from Table 2, the reaction of aromatic aldehydes with malononitrile and 1,3-diketones at room temperature under solvent-free conditions provided the corresponding 4*H*-benzo[*b*]pyran derivatives in good yields. The results presented in Table 2 indicate that aldehydes bearing electron-withdrawing groups react more quickly than their electron-donating aldehyde counterparts. For example, aromatic aldehydes such as 4-chloro-, 4-nitro-, and 4-bromobenzaldehydes react quickly with high product yields in comparison to 4-hydroxy-, 4-methyl-, and 4-methoxybenzaldehyde derivatives. The yield of 4*H*-benzo[*b*]pyrans bearing group at the *ortho* position on the aromatic ring is lower than that of the 4*H*-benzo[*b*]pyrans bearing group at the *para* position on the aromatic ring (Scheme 1; Table 2).

**Table 2:** Synthesis of 4*H*-benzo[*b*]pyran derivatives catalyzed by FeNi_3_/SiO_2_/HPG MNP

Entry	R	1,3-Diketone	Product	Time/min	Yield/%^a,b^	M.p. (obs)/°C	M.p. (lit)/°C
1	C_6_H_5_	**1a**	**4a**	30	97	228–230	224 [25]
2	4-ClC_6_H_4_	**1a**	**4b**	30	96	207–209	209–211 [25]
3	2-ClC_6_H_4_	**1a**	**4c**	35	94	210–212	214–215 [25]
4	4-MeC_6_H_4_	**1a**	**4d**	40	90	228–230	223–225 [25]
5	4-NO_2_C_6_H_4_	**1a**	**4e**	30	94	183–186	179–180 [25]
6	2-NO_2_C_6_H_4_	**1a**	**4f**	35	92	217–220	222–223 [26]
7	4-BrC_6_H_4_	**1a**	**4** **g**	30	95	200–202	203–205 [26]
8	2-BrC_6_H_4_	**1a**	**4h**	35	93	152–154	150–152 [26]
9	4-MeOC_6_H_4_	**1a**	**4i**	40	91	201–202	199–201 [25]
10	4-HOC_6_H_4_	**1a**	**4j**	40	89	201–204	206–208 [25]
11	4-FC_6_H_4_	**1a**	**4k**	35	97	188–190	192–194 [27]
12	4-(Me_2_N)C_6_H_4_	**1a**	**4l**	40	88	199–201	198–200 [25]
13	C_6_H_5_	**1b**	**4m**	30	95	235–237	239–241 [28]
14	4-ClC_6_H_4_	**1b**	**4n**	30	97	222–224	226–229 [28]
15	2-ClC_6_H_4_	**1b**	**4o**	30	95	213–215	210–212 [28]
16	4-MeC_6_H_4_	**1b**	**4p**	40	93	224–226	223–225 [28]
17	4-NO_2_C_6_H_4_	**1b**	**4q**	35	95	235–236	234–236 [28]
18	2-NO_2_C_6_H_4_	**1b**	**4r**	35	92	191–193	196–198 [28]
19	4-MeOC_6_H_4_	**1b**	**4s**	40	89	188–190	193–195 [28]
20	4-HOC_6_H_4_	**1b**	**4t**	40	88	229–232	234–236 [28]
21	4-FC_6_H_4_	**1b**	**4u**	30	96	217–220	213–215 [28]
22	4-(Me_2_N)C_6_H_4_	**1b**	**4v**	40	89	173–175	168–170 [28]
23	C_6_H_5_	**1c**	**4w**	35	96	193–195	194–196 [27]
24	4-ClC_6_H_4_	**1c**	**4x**	30	97	173–175	175–177 [27]
25	4-MeC_6_H_4_	**1c**	**4y**	40	92	180–182	177–179 [27]
26	4-MeOC_6_H_4_	**1c**	**4z**	40	91	140–142	137–139 [27]
27	4-NO_2_C_6_H_4_	**1c**	**4a′**	30	98	183–185	180–183 [27]

^a^Reaction condition: benzaldehyde derivatives (1 mmol), 1,3-diketones (1 mmol), malononitrile (1 mmol), 0.001 g FeNi_3_/SiO_2_/HPG MNP at room temperature under solvent-free conditions

^b^Yield refers to isolated product

On the other hand, when benzyl cyanide was treated as a substitute for malononitrile in this reaction under similar conditions, not only was a highly prolonged time required, but the products were different. The spectroscopic data of the products confirmed that these structures belong to octahydroxanthene (Scheme 2).

**Figure:**
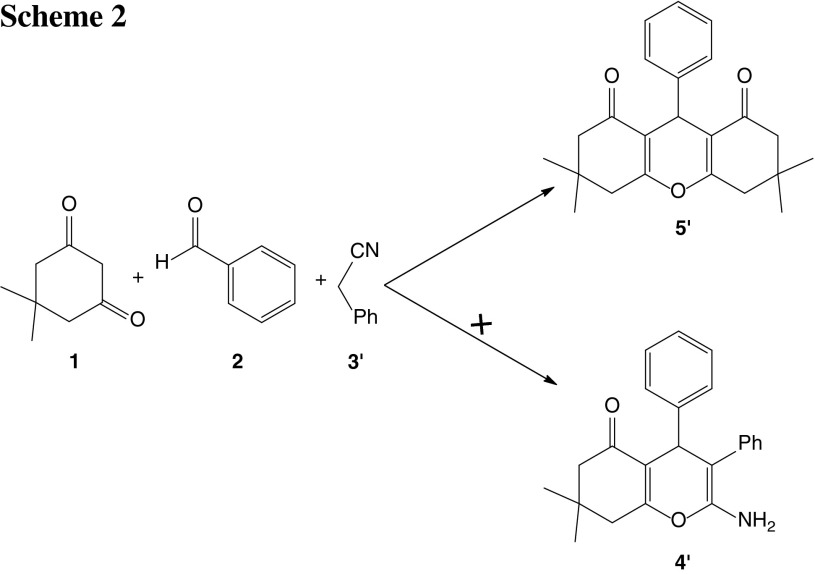

In comparison with other catalysts employed for the synthesis of 4*H*-benzo[*b*]pyran from malononitrile, benzaldehyde, and dimedone, FeNi_3_/SiO_2_/HPG MNP showed a much higher catalytic activity in terms of a very much shorter reaction time and mild conditions (Table 3).

**Table 3:** Comparison of the catalytic efficiency of FeNi_3_/SiO_2_/HPG MNP with that of other catalysts

Entry	Catalyst	Condition	Solvent	Amount catalyst/g	Time/min	Yield/%^a^
1	Yb(PFO)_3_	60 °C	EtOH	1.8	300	90 [17]
2	Tetramethylammonium hydroxide	r.t.	H_2_O	0.09	30–120	81 [18]
3	Na_2_SeO_4_	Reflux	H_2_O/EtOH	0.1	60	97 [19]
4	LiBr	Reflux	H_2_O	8.7	15	95 [20]
5	NaBr	20 °C	*n*-PrOH	0.01	25	84 [21]
6	MgO	r.t.	H_2_O	0.02	30	75 [22]
7	NaBr	MW	–	0.042	10	95 [23]
8	SB-DABCO	r.t.	EtOH	0.06	35	96 [24]
9	FeNi_3_/SiO_2_/HPG MNP	r.t.	None	0.001	30	97

^a^Isolated yield, conditions: malononitrile (1 mmol), benzaldehyde (1 mmol), and dimedone (1 mmol)

To further explore the potential of this MNP catalyst for heterocyclic synthesis, we investigated one-pot reactions involving aromatic aldehydes, malononitrile, ethyl acetoacetate, and hydrazine hydrate and obtained pyranopyrazoles in excellent yields (Scheme 3; Table 4). This methodology was evaluated using a variety of different substituted aromatic aldehydes in the presence of magnetic nanocatalyst under similar conditions. Aromatic aldehydes, carrying either electron-withdrawing or electron-donating substituents, afforded high yields of products with high purity; the results are presented in Table 4. The four-component cyclocondensation reaction proceeded smoothly and was completed in 35–50 min.

**Figure:**
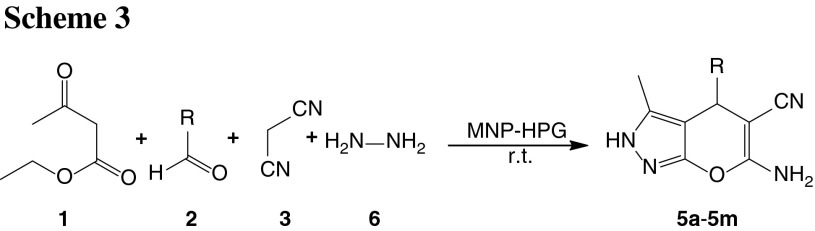

**Table 4:** Synthesis of pyranopyrazoles from various aromatic aldehydes, malononitrile, ethyl acetoacetate, and hydrazine hydrate in the presence of magnetic nanocatalyst at room temperature under solvent-free conditions

Product^a^	R	Yield/%^b^	Time/min	M.p. (obs)/°C	M.p. (lit)/°C
**5a**	C_6_H_5_	94	35	244	245–246 [29]
**5b**	4-ClC_6_H_4_	95	35	233	234–235 [30]
**5c**	2-ClC_6_H_4_	91	45	245	245–246 [31]
**5d**	4-MeC_6_H_4_	93	50	197	197–198 [32]
**5e**	4-NO_2_C_6_H_4_	92	35	250	251–252 [29]
**5f**	2-NO_2_C_6_H_4_	90	40	240	242–243 [32]
**5g**	4-BrC_6_H_4_	92	45	249	249–250 [32]
**5h**	3-BrC_6_H_4_	88	50	233	223–224 [29]
**5i**	2-MeOC_6_H_4_	91	50	250	252–253 [32]
**5j**	4-MeOC_6_H_4_	92	45	212	212–213 [33]
**5k**	4-HOC_6_H_4_	88	45	224	223–224 [33]
**5l**	4-FC_6_H_4_	90	40	246	247–248 [32]
**5m**	4-(Me_2_N)C_6_H_4_	92	50	220	219–220 [32]

^a^All products were identified and characterized by comparison with authentic samples

^b^Yield refers to isolated product

## Conclusion

In conclusion, we have developed current important areas in the heterogenization of HPG—a rapidly developing research area. The main objectives are to develop room-temperature, solvent-free conditions, a rapid (immediate) and easy immobilization technique, and low-cost precursors for the preparation of highly active and stable MPs with high densities of functional groups. Furthermore, applying the exciting new area of magnetic particles that are intrinsically not magnetic, but can be magnetized readily by an external magnet, can have a positive effect on high activity on the one hand and separation and recycling on the other.

## Experimental

Chemical materials were purchased from Fluka (Buchs, Switzerland) and Merck (Darmstadt, Germany) in high purity. Melting points were determined in open capillaries using an Electrothermal 9100 apparatus (http://www.electrothermal.com). Morphology was analyzed using high-resolution transmission electron microscopy (HRTEM) on a JEOL transmission electron microscope (http://www.jeol.com) operating at 200 kV. Powder X-ray diffraction data was obtained using Bruker D8 Advance model with Cu-Kα radiation. The thermogravimetric analysis (TGA) was carried out on a NETZSCH STA449F3 (http://www.netzsch-thermal-analysis.com) at a heating rate of 10 °C min^−1^ under nitrogen. The magnetic measurement was carried out in a vibrating sample magnetometer (VSM) (4 inch, Daghigh Meghnatis Kashan, Kashan, Iran) at room temperature. NMR spectra were recorded in DMSO-*d*_*6*_ on a Bruker Avance DRX-400 MHz instrument spectrometer (http://www.bruker.com/) using tetramethylsilane (TMS) as internal standard. IR spectra were recorded on a Perkin Elmer 781 (http://www.perkinelmer.com/). Mass spectra were recorded on Shimadzu GCMS-QP5050 mass spectrometer (Shimadzu, Tokyo, Japan). The purity determination of the products and reaction monitoring were accomplished by thin layer chromatography (TLC) on silica gel polygram SILG/UV 254 plates.

### Synthesis of FeNi_3_ MNPs

FeCl_2_·4H_2_O (1.72 g) and 4.72 g NiCl_2_·6H_2_O were dissolved in 80 cm^3^ deaerated highly purified water contained in a three-neck flask with vigorous stirring (800 rpm) under nitrogen. As the temperature was elevated to 80 °C, 10 cm^3^ ammonium hydroxide was added drop by drop, and the reaction was maintained for 30 min. The black product was separated by placing the vessel on a permanent magnet and the supernatant was decanted. The black precipitate was washed six times with highly purified water to remove unreacted chemicals, then the black product FeNi_3_ was dried under vacuum.

### Synthesis of FeNi_3_/SiO_2_ MNPs

First, a mixture of 100 cm^3^ ethanol and 20 cm^3^ distilled water was added to 1 g magnetite nanoparticles, and the resulting dispersion was sonicated for 10 min. After adding 2.5 cm^3^ ammonia water, 2 cm^3^ tetraethyl orthosilicate (TEOS) was added to the reaction solution. The resulting dispersion was mechanically stirred continuously for 20 h at room temperature. The magnetic FeNi_3_/SiO_2_ nanoparticles were collected by magnetic separation and washed with ethanol and deionized water in sequence.

### Synthesis of FeNi_3_/SiO_2_/HPG MNPs

For synthesis of FeNi_3_/SiO_2_/HPG MNPs, 2 mmol FeNi_3_/SiO_2_ MNPs were dispersed in a mixture of 80 cm^3^ toluene and 1.0 mmol potassium methanolate (CH_3_OK), followed by the addition of 10 cm^3^ anhydrous dioxane. Glycidol (2.0 g) was added dropwise over a period of 15 h. After vigorous stirring for 2 h, the final suspension was repeatedly washed, filtered several times, and air-dried at 60 °C.

### General procedure for the synthesis of 4*H*-benzo[*b*]pyran

A mixture of aromatic aldehyde (1 mmol), dimedone (1 mmol), malononitrile (1 mmol), and 0.001 g FeNi_3_/SiO_2_/HPG MNP was stirred at room temperature under solvent-free conditions for the appropriate time (Table 2). Upon completion (the progress of the reaction was monitored by TLC), EtOH was added to the reaction mixture and the FeNi_3_/SiO_2_/HPG MNP was separated by external magnet. The solvent was then removed from solution under reduced pressure and the resulting product purified by recrystallization using ethanol.

### General procedure for the synthesis of pyranopyrazoles

A mixture of ethyl acetoacetate (1 mmol), hydrazine hydrate (1 mmol), malononitrile (1 mmol), aldehyde (1 mmol), and 0.001 g FeNi_3_/SiO_2_/HPG MNPs was stirred at room temperature under solvent-free conditions for the appropriate time (Table 4). Upon completion (the progress of the reaction was monitored by TLC), EtOH was added to the reaction mixture and the FeNi_3_/SiO_2_/HPG MNPs was separated by external magnet. The solvent was then removed from solution under reduced pressure and the resulting product purified by recrystallization using ethanol.
